# An Impact Localization Solution Using Embedded Intelligence—Methodology and Experimental Verification via a Resource-Constrained IoT Device †

**DOI:** 10.3390/s23020896

**Published:** 2023-01-12

**Authors:** Ioannis Katsidimas, Vassilis Kostopoulos, Thanasis Kotzakolios, Sotiris E. Nikoletseas, Stefanos H. Panagiotou, Constantinos Tsakonas

**Affiliations:** 1 Computer Engineering and Informatics Department, University of Patras, 26504 Patras, Greece; 2 Mechanical Engineering and Aeronautics Department, University of Patras, 26504 Patras, Greece; 3 Computer Technology Institute and Press “Diophantus”, 26504 Patras, Greece

**Keywords:** resource-constrained IoT, TinyML, structural health monitoring, PZT sensors, PMMA plate, industrial WSN

## Abstract

Recent advances both in hardware and software have facilitated the embedded intelligence (EI) research field, and enabled machine learning and decision-making integration in resource-scarce IoT devices and systems, realizing “conscious” and self-explanatory objects (smart objects). In the context of the broad use of WSNs in advanced IoT applications, this is the first work to provide an extreme-edge system, to address structural health monitoring (SHM) on polymethyl methacrylate (PPMA) thin-plate. To the best of our knowledge, state-of-the-art solutions primarily utilize impact positioning methods based on the time of arrival of the stress wave, while in the last decade machine learning data analysis has been performed, by more expensive and resource-abundant equipment than general/development purpose IoT devices, both for the collection and the inference stages of the monitoring system. In contrast to the existing systems, we propose a methodology and a system, implemented by a low-cost device, with the benefit of performing an online and on-device impact localization service from an agnostic perspective, regarding the material and the sensors’ location (as none of those attributes are used). Thus, a design of experiments and the corresponding methodology to build an experimental time-series dataset for impact detection and localization is proposed, using ceramic piezoelectric transducers (PZTs). The system is excited with a steel ball, varying the height from which it is released. Based on TinyML technology for embedding intelligence in low-power devices, we implement and validate random forest and shallow neural network models to localize in real-time (less than 400 ms latency) any occurring impacts on the structure, achieving higher than 90% accuracy.

## 1. Introduction

### 1.1. TinyML

Recent advances in algorithms, hardware and software have facilitated the realization of performing on-device sensor data analytics, namely TinyML [1] (also known as extreme edge ML, embedded ML or embedded intelligence), in low power and limited resources IoT devices. Mainly focusing on, but not limited to, applications that are related to (i) computer vision (ii) audio and speech processing, (iii) natural language processing, and (iv) activity recognition, has set a strong basis for a lot more potential modern systems that promote principles such as decentralization and cost effectiveness. TinyML can also be characterised as the epitome of utilisation and resources availability, as it successfully demonstrates the most effective use of models and resources, to achieve an acceptable performance to the service provided. TinyML has also contributed to the realization of novel paradigms such as smart objects and smart sensing, presenting a new era in the Internet of Things (IoT) research area, where embedded devices present advanced intelligence capabilities, resulting in the Artificial Intelligence of Things (AIoT) or Intelligent IoT (IIoT).

Successful deployment of such systems requires addressing the scarcity of computational and energy resources for real-time (or near real-time) calculations. Such solutions inherently provide several advantages, namely scalability, and easy installation (due to small physical dimensions), less transmissions and no latency (due to local process), and data protection (as the readings are not exposed out of the device). However, to achieve self-reflection and enhance the system’s performance parameters, the whole process pipeline from the methodology and the data collection, to the final deployment of the model on the device, must be complete and well designed, while various challenges must be addressed including computational and memory limited resources, and low energy consumption to perform real-time (or near real-time) applications.

### 1.2. Structural Health Monitoring

Structural health monitoring (SHM) plays a vital role in decision-making concerning maintenance operations and strategies, making it a very wide application subject with many challenges to apply sensing and processing capabilities.

It covers a massive area of interest as it improves reliability, life, quality control, productivity and safety. Thus, different classes can be found to taxonomize each application based on the construction and the corresponding requirements; (i) machine condition monitoring, (ii) global monitoring of large structures, (iii) large areas monitoring, and (iv) local monitoring [2]. These groups of applications present a common vertical considering damage classifier into four distinct levels, namely the damage detection, localization, assessment and prediction through the remaining life estimation. In some cases we may also see a self healing layer that can be applied, depending on the use case. Impact detection and localization refer to the first two levels, and can be used to make a prognosis on the potential existence of damage.

The core element to a modern digitalized SHM system is sensing, which in most cases includes vibroacoustic, piezoelectric, stain gauges, fiber optics, cameras (for image analysis), fluid (lubricant, oil) pressure and consumption, accelerometers, and thermography sensors. Vibration-like sensors are the most widely used in SHM systems as they enable vibration detection that results in an equivalent electrical signal that feeds a processing unit. Vibration analysis is an accepted and reliable method for monitoring the operation and performance of the structures, while its transparent adoption without any process interference offers a sustainable monitoring solution. Most common signal processing methods include Fourier transformation (and its variants such as fast and short-time), statistical analysis over time-series, Cohen’s class, wavelet transform, Hilbert–Huang transformation, neural networks (NN), Bayesian classifiers and further hybrid approaches [3]. Finally, there are typically two approaches that mainly attempt to deal with SHM problems [4]; (i) knowledge-based or model-driven models, which in some cases either neglect realistic and real life conditions or make some convenient assumptions, and (ii) data-based models, which suffer from sufficient data availability, as running an experiment multiple times is costly in time, equipment and personnel.

Modern and critical paradigms such as the circular economy and additive manufacturing, set plastic parts and items in a leading role due to the many advantages that natively offer and facilitate desired principles, such as cost-effectiveness, reusability, recycling, less material waste, mass customisation capabilities, etc. However, these benefits come together with challenges, namely condition monitoring, tracking and tracing that must be addressed for transparent integration both in industry and everyday life. Integrated TinyML seems a quite promising technology to address some of these challenges in a transparent and decentralized way, offering technical assistance in decision-making using processed information that is produced autonomously and without further conflicts and impedances.

### 1.3. Aim and Motivation

In this work, we investigate impact loads on a transparent poly-methyl-methacrylate (PMMA) thin-plate structure. PMMA is a synthetic polymer from the methyl methacrylate monomer and it is widely used instead of inorganic glass, due to its high impact strength and low, as well as easy, processing. The first major application of the polymer took place during World War II, when PMMA was used as aircraft windows and bubble canopies for gun turrets. Nowadays, PMMA is used in car windows, motorcycle windshields, interior and exterior panels, fenders, etc. It is also used for ship windows (salt resistance) and aviation purposes. Furthermore, PMMA is used in solar panels due to its mechanical strength, compatibility, and optical clarity [5,6,7]. From the aforementioned applications, it is concluded that PMMA is used where operational loads are combined with impact loads either caused by accidents (tool drops, etc.) or due to environment (hail impacts). In plastics, impacts often cause critical damages that under continuous cyclic loads lead to internal crack propagation and further weakening of the component until it experiences critical failure under regular loads [8]. Knowing the location of the impact is a key factor in successful examination of the component because traditional inspection methods inspect the structure locally [9]. Additionally, because thin surfaces are the most heavily exposed to impacts, the majority of experimental work concerning impacts used plate structures as specimens. To this end, it is crucial that a system exists near a PMMA structure, capable to perform impact monitoring tasks such as the detection of the location of an impact and thus providing information about failure-prone areas and decay in its condition.

We regard our paper as a significant work towards the broad use of WSNs in advanced IoT applications, as we aim to integrate effectively the characteristic resource-constrained nature of sensors into an industrial application characterized by strict real-time specifications (e.g., systems that are installed on the road surface, demonstrated in [10], and for infrastructure monitoring purposes [11], that also need sustainable SHM tools for the structure itself, to meet their autonomy requirements). Our approach targets several problems and needs in Industrial IoT applications such as the low-latency, real-time information exchange and decision-making, the concerns for security and privacy, the limited bandwidth for data sharing as well as the unreliable performance of the wireless channel into harsh production environments. Thus, we propose an online, on-device data processing solution that minimizes external communications, decreases the costs and ensures system flexibility, mobility, safety and security.

To the best of our knowledge, this is the first work in which a TinyML-based SHM system is implemented in plastic (PMMA) structures. We aim to design and implement a real-time impact detection and localization model based on TinyML principles, that contributes to the continuous monitoring of impacts over a mechanical system made by PMMA. The main contributions of this work are summarized as follows:

- On-device intelligence (powered by TinyML) to solve the impact localization problem of a thin plate, by introducing two models that perform effectively with respect to TinyML principles.
- Real-time data collection methodology with an Arduino 33 BLE MCU (in contrast to the literature that uses oscilloscopes), that achieves and demonstrates high sampling frequency (100 KHz) and extreme low latency (8.5 μs).
- Sharing a new publicly available dataset, denoted as “Impact Events”, that contains PZT sensor measurements concerning low-velocity, low-energy impact events in a thin PMMA plate [12].

Roadmap of the paper. The rest of this paper is organized as follows. Section 2 elaborates on the related work in ML-driven impact localization and industrial TinyML research and afterwards in Section 3 we present the methodology of our work, by defining the thin plate and sensors setup, the design and setup of our experiments and the associated monitoring system. Moreover, in Section 4, we describe our approach for a resource-constrained sensing solution, the contructed dataset and feature extraction process, and in Section 5, the models and the rationale behind their selection are presented. Section 6 contains the outcomes and technical discussion of the experimental results and, finally, in Section 7 we summarize the subject of the work and report our next steps for further exploitation.

## 2. Related Work

To the best of our knowledge, there is a gap in the literature concerning the availability of public SHM datasets and impact localization methodologies that deploy TinyML models in resource-constrained edge devices. Therefore, in this section we present some research works which can be considered as the most related to the current paper. The section is divided in three parts, covering related work in plate structure impact localization based on machine learning (ML) techniques and data from piezoelectric sensors (PZTs), general research related to industrial TinyML applications and currently available SHM datasets.

### 2.1. Impact Localization

Dipietrangelo et al. [13] studied the low speed impact localization problem over a rectangular, isotropic thin structure made of aluminum, while the impact was produced by a steel sphere, to finally provide two supervised machine learning models, using a shallow neural network (SNN) and polynomial regression (PR). The authors used piezoelectric experimental data from 4 sensors and the monitoring procedure was supported by standard equipment (oscilloscope, amplifier). The results were based on a mean radial error metric (MRE), achieving quite impressive and acceptable accuracy (SNN with 1.2 mm and PR with 1.5 mm).

For the same experimental setup, Balasubramanian et al. [14] developed and validated a convolutional neural network (CNN), a long short-term memory (LSTM) network, and an artificial neural network (ANN), with respect to mean absolute error (MAE) metric. Although, the accuracy of these networks is reduced compared to the SNN (when being shallower and wider), are found to be more robust in cases where noise is present. In particular, the MAE of ANN was 22 mm (11% accuracy reduction with noise), CNN was 31 (2% accuracy reduction with noise), and LSTM was 25 mm (7% accuracy reduction with noise). Closer to our work, in terms of the material, as the experiments consider carbon fiber reinforced plastic (CFRP), Datta et al. [15] solved low-velocity impact (LVI) events localization, by estimating X, Y coordinates, alongside the corresponding force of the impact. To this end, the authors proposed a least square support vector regression-based algorithm that performs over two types of data that are produced from fiber optic sensors and resistance strain gauges. For the experimental test setup, special equipment (with abundant resources) was used for the data collection, able to provide integrated data acquisition, signal conditioning and a list of different options regarding sampling rates.

Hesser et al. [16] used knowledge-based piezoelectric data to train and develop ANN and support vector machine (SVM) models to solve LVI localization problem caused by steel ball over an aluminum thin-plate. In particular, training data was produced by simulations that the authors managed to implement via a finite element model. However, the validation was performed over experimental data (acquired by oscilloscope instrument and a data logger). The results showed that the ANN achieved 1.8 mm mean value of the error estimation when the impact occurs inside the area that the PZT sensors form, and 2.6 mm when it occurs outside, while the SVM achieves 3 mm and 14.3 mm, respectively. Tabian et al. [17] developed a metamodel using convolutional neural networks (CNN) and passive sensing to detect, localize, and characterize impacts on complex composite structures. By using raw data from a network of PZT sensors and transforming it into 2D images, the model was able to accurately predict impacts on similar locations that it had been trained on, with prediction accuracy ranging from 94.3 to 100%. The metamodel’s scalability was also demonstrated by its ability to accurately predict impacts on locations outside of the training region, with prediction accuracy exceeding 95% in most cases.

In [18], Jung et al. developed a CNN-based model that can identify impacts on composite structures by analyzing PZT signals. They converted the signals into image data using discrete wavelet transform and used data augmentation to increase the amount of training data. When data augmentation was not used, the model’s error in predicting impacts was large (up to 27.5%), but using data augmentation to double the training data size reduced the maximum and average prediction errors to 13.0% and 5.7%, respectively.

Karmakov et al. [19] introduced a new method for classifying impacts on composite structures using self-attention, a key component of the transformer neural network. They transformed raw time series data from piezoelectric sensors into Fourier transform data and compared the performance of the transformer network to a CNN. Both models were able to accurately classify the energy of steel impacts with 100% accuracy using as few as 378 samples, and the transformer required less computational power for training and prediction compared to the CNN.

### 2.2. TinyML in Industrial Applications

Zonzini et al. [20] studied the application of TinyML for vibration-based structural health monitoring scenarios. The authors experimented with the one class classifier neural network (OCCNN) into the Arduino Nano 33 BLE Sense (Arduino LLC, Italy), which is the same resource-constrained device that we also use in the present study. The authors used data from the Z24 bridge use case to benchmark the OCCN and they achieved an average accuracy and precision of 95% and 94%, respectively. In [21], different NN architectures are benchmarked, namely CNN, LSTM, CNN LSTM, GRU, and CNN GRU, for estimating maximum capacity of Li-Ion batteries using Li-Ion battery datasets provided by NASA. The authors compare the STM32Cube.AI and TensorFlow Lite for microcontrollers (TFLM) tools, demonstrating that the former outperforms TFLM in terms of average inference time [ms], RAM size including inputs buffer [KB], and Flash size [KB]. TinyML for remaining useful life prognosis was studied in the work of Athanasakis et al. [22]. The authors used the popular C-MAPSS NASA dataset that is comprised of simulation time series sensor data and used the STMF767ZI microcontroller (STMicroelectronics, Switzerland) along with X-CUBE-AI tool, to predict the RUL of turbofan engines. They optimized several models such as Long Short Term Memory (LSTM), Convolutional Neural Network (CNN), XGBoost and random forest. Concerning the accuracy and model footprint metrics, the authors report that the quantized CNN models can reach as low as 26 KB of Flash and 9 KB of RAM memory that leads to an average 10% quality loss in the RMSE metric for embedded devices.

Ren et al. [23], propose a novel system called TinyOL (TinyML with online-learning), which enables incremental on-device training on streaming data. TinyOL is based on the concept of online learning and is suitable for constrained IoT devices. They experimented with TinyOL under supervised and unsupervised setups using an autoencoder neural network for multi-anomaly classification, deployed to an Arduino Nano 33 BLE MCU board using TFLite Micro library and validated in a USB fan. In Bratu et al. [24], a low-power unsupervised learning solution is proposed for the detection of anomalies in the vibration patterns of bearings. An autoencoder takes as input the median absolute deviation of each measurement set produced by an accelerometer, and then a classifier compares the values provided by the output with values that are known to be normal vibration patterns. The authors report that the models deployed in an ESP32 board with TensorFlow Lite, achieve up to 93.42% accuracy. Lastly, Funk et al. [25], propose a lightweight direct inverse NN-based control approach for controlling the angular speed of a permanent magnet DC motor, which runs on a tiny Arm Cortex-M0 microcontroller with only 4 kB of RAM. The NN can be trained and executed on a tiny ARM Cortex-M0 microcontroller and all necessary neural network functions (activation functions and derivatives, feedforward, backpropagation, batch-gradient calculation and Adam) were implemented from scratch in embedded C.

### 2.3. Limitations of Existing Datasets

Despite the large number of research works on SHM, the vast majority do not share their datasets. Azimi et al. [26], provide an extensive list of those publications that do share their datasets (with vibration and mostly vision-based data) that have been recently used in deep learning-based SHM. However, for impact detection and localization in plate structures with PZT sensors, which is also a very well studied problem in the literature (for example, [13,14,15,27,28]), there are no openly available datasets.

Regarding some indicative open access datasets (for any SHM-related problem), Bechhoefer et al. [29] have published data for wind turbine high-speed bearing prognosis and also Figueiredo et al. [30] from Los Alamos National Lab have published standardized datasets intending to familiarise users with feature extraction and statistical modelling for feature classification in the context of SHM. Teloli et al. [31] have a published dataset named UNESP-BERT for bolted joint SHM based on vibration tests. Finally, Marzani et al. [32], have made available a dataset for benchmarking guided waves on a composite full-scale outer section of an aircraft wing. However, by utilizing the aforementioned datasets, the algorithms that can be trained focus on detecting, and sometimes (but not always), locating damage (instead of impact) but they do not consider environmental and operational factors, and just rely on specific damages (change in geometry, material) that occur in the structure.

## 3. Methodology

In this section, we provide the methodology that was followed to execute the experiments and create an Impact Events dataset. First, we describe the design of the thin plate structure. Afterward, we provide the details for the design of experiments, how and why they were performed and then we outline the procedure and challenges to realize the data acquisition with the edge-sensing device.

### 3.1. Thin Plate and Sensors Setup

**Figure 1:**
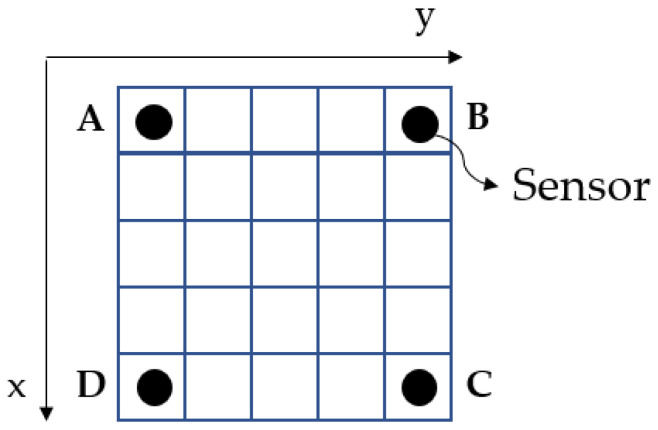
Thin plate model with the sensors and the impact localization areas. Letters A, B, C, D correspond to the respective sensors, as they are denoted later in the paper as SensorA, SensorB, etc.

The structure that is used is a thin PMMA plate *P* with dimensions of 300 mm × 300 mm and 4 mm thickness and with four piezoelectric transducers (PZTs) bonded at the corners, as shown in Figure 1. The grid is separated in a grid of 25 identical 60 mm × 60 mm square tiles, to allow the modeling of impact localization problems with classification approaches. However, since the grid tiles are essentially derived from the *X* and *Y*-coordinates of the impact, the number and size of tiles can be arbitrarily formulated to fewer or more labels depending on the research purposes.

The set of the squares is denoted as $Q=\{q_{1,1},\;q_{1,2},\ldots ,\;q_{1,5},\;q_{2,1},\;q_{2,2},\ldots ,\;q_{2,5},\;q_{3,1},\ldots ,\;q_{5,1},\ldots ,\;q_{5,5}\}$ where *x* and *y* at $q_{x,y}$ is the row and column square, respectively, thus PZT A is deployed at $q_{1,1}$, PZT B at $q_{1,5}$, PZT C at $q_{5,5}$, and PZT D at $q_{5,1}$ (and they are denoted as sensor [A|B|C|D] in the following sections).

**Figure 2:**
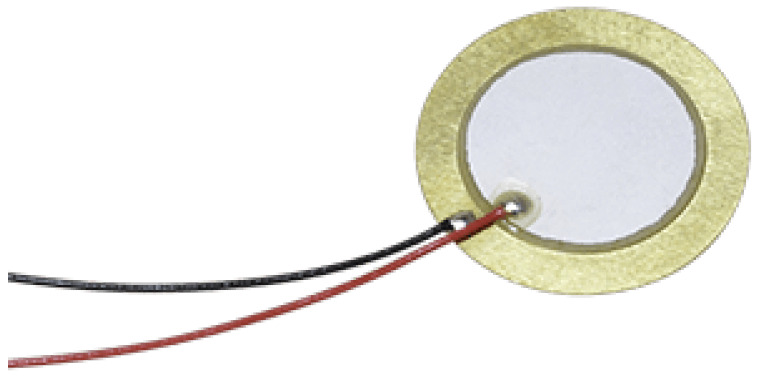
Ceramic piezoelectric transducer CEB-35D26.

In Table 1 and Figure 2, we provide the attributes of the ceramic piezoelectric transducer CEB-35D26 (Mouser Electronics, Mansfield, TX, USA) [33] that was used and below we provide the formula for correlating the force applied to the output voltage of the piezoelectric sensor.

(1) $$F=V\times d_{33}\times C$$

where

*F* = Applied force
*V* = Output voltage
*d*_33_ = Piezoelectric constant
*C* = Stiffness factor of ceramic

For the CEB-35D26, *d*_33_ = 460 pC/N and *C* = 60 × 10^9^ N/m^2^.

### 3.2. Design and Execution of Experiments

This paper introduces a real-time solution, to accomplish the impact localization at the edge utilizing TinyML models. Thus, we consider the following problem definition.

**Impact Localisation Multiclass Classification Problem:** Given a plate *P* and a set of piezoelectric sensors *S*, determine the area *Q* in which an impact event occurs.

The data acquisition portion of the SHM process involves selecting the excitation methods—the sensor type and number, and locations—and the data acquisition/storage/ processing/transmittal hardware. It is important to associate the sensors’ response with the parameters defining the ball impact. This can be accomplished with a methodology called experiment design or design of experiments (DOE), which generates test sequences that correspond to the impact experiments. The test sequences consist of operating points of input variables of the system, which are the x-coordinate, the y-coordinate, and the fall height. The design is maximized so that the model can provide an accurate prediction for the system’s response given the sensors’ response.

**Figure 3:**
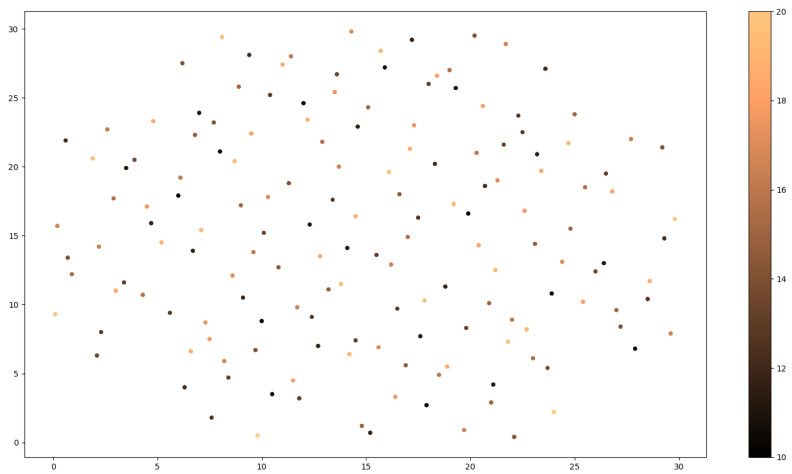
Two-dimensional representation of impact coordinates and fall height combinations (with different colors), based on Sobol sequences.

Generally, the design of experiments is a systematic, efficient and effective way of a method that enables the study of relationships between multiple input variables (or factors) and key output variables (or responses). It is a structured approach to collecting data and making discoveries. Our design of experiments is divided into categories based on the criterion by which the sequences of data and the knowledge we have about the system being studied. The two major categories are space-filling and optimal DOEs. This particular problem was treated as an unknown system. Therefore, a space-filling DOE was selected, and more specifically the Sobol sequence. Sobol sequences, also known as $LP_{T}$ sequences, are categorized as a space-filling design. This design is a quasi-random sequence, in which the test sequences generated are randomly planned in the design boundary. The design boundaries are specified by the user regarding each input variable. However, it is possible for physical restrictions to exist, such as the geometric properties of the structure, which is visible in our study. Thus the boundaries set for the *x* and *y* coordinates are in the range [0, 300] as the dimensions of the plate, whereas the height boundary is arbitrarily set. The test sequences are iteratively generated in a uniform distribution, by utilizing the primitive polynomials over a Galois Field and gray code encoding. The most fascinating characteristic of Sobol sequences is that they generate test sequences in a high degree of scattering while avoiding overlapping of previous test sequences. The deterministic quasi-random characteristic of Sobol sequences enables progressive augmentation of the test sequences [34,35]. In Figure 3, we provide the 2D representation of impact coordinates and fall height combinations based on Sobol sequences.

**Figure 4:**
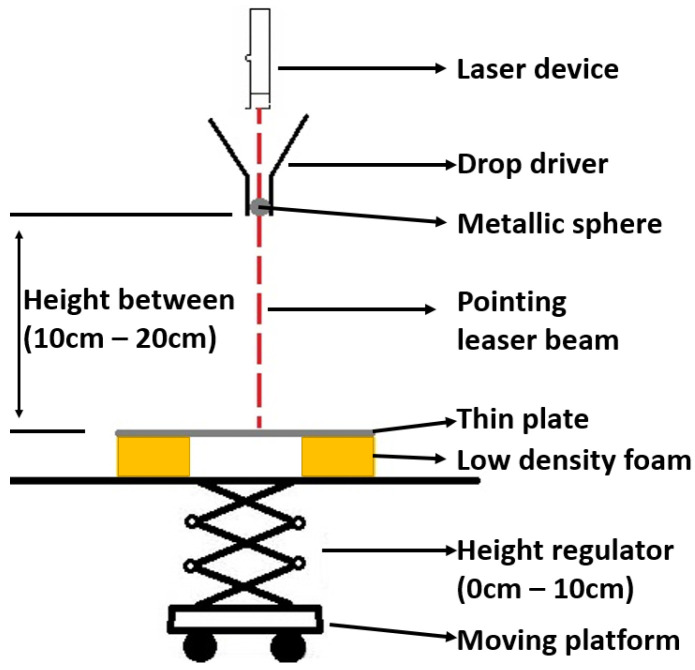
Visualization of the steel ball drop setup.

In Figure 4, we depict the setup for dropping the steel ball. Specifically, the impact event is produced by a steel ball *B* (9.5 mm diameter, 3.53 g weight), which is released from a height distance that varies from 10 to 20 cm, with 0.5 cm interval, from the *P*, and performs a free-fall (the initial state of the sphere has zero acceleration with the help of the drop driver). The laser device and the drop driver are statically mounted and the change of impact location takes places by moving the plate in the desired *X*- and *Y*-coordinates, which are verified using the laser beam. We also note that in the experimental procedure, we do not perform impact events in the square tiles where the sensors are included (i.e., in $q_{1,1}$, $q_{1,5}$, $q_{5,1}$, $q_{5,5}$), as the latter return extreme and noisy values. Overall, each experiment is repeated at least three times in order to enhance the dataset and ensure the robustness of the experiment, resulting in a total of 771 experiments (multiple repetitions for 159 distinct impacts).

### 3.3. Monitoring System

**Figure 5:**
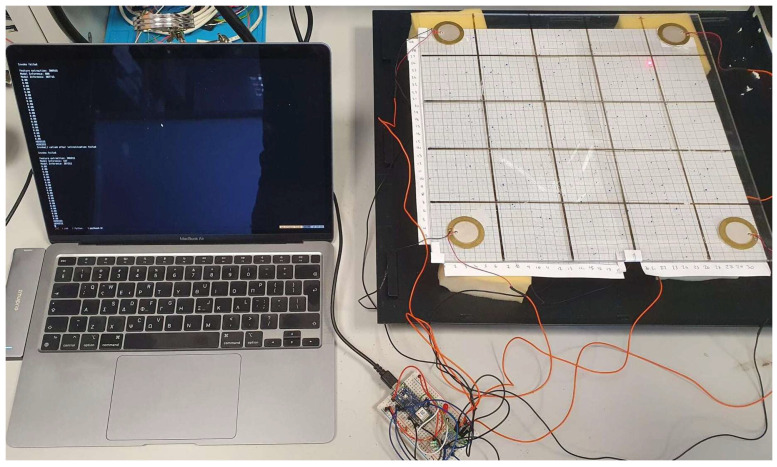
Image of the experimental plate setup.

**Figure 6:**
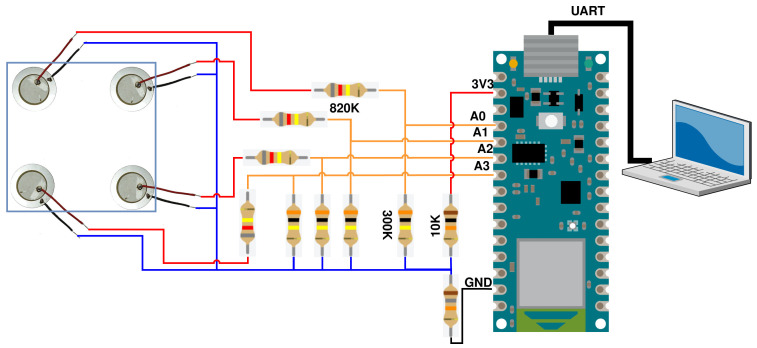
PZT modules connected to Arduino NANO 33 BLE.

In order to monitor the structure, acquire the propagated wave, and make the impact localization, the piezoelectric sensors are bonded to the structure and connected to a microcontroller that is responsible to perform impact localization services. Figure 5 and Figure 6 depict this sensing setup. The laptop is used to store the data (for ML training) that the Arduino device reads from the piezoelectric sensors and the impact identification model runs on the Arduino device.

The controller is an Arduino NANO 33 BLE (Arduino LLC, Roma, Italy), and it was selected due to the memory capacity and processor speed it provides. It is also one of the suggested devices for TinyML applications, while being acceptable to both industry and the research community. Moreover, it is compatible with the most state-of-the-art TinyML frameworks. The voltage divider between the sensors and the device shifts the reference voltage of the idle state to a value greater than zero. This further enables the data acquisition, since as soon as the impact occurs, the structure flexes, producing positive and negative output voltage by the PZTs.

For the given and related problems, different types of material and thickness of the plate will result in different eigenfrequency, thus the sampling rate should be changed accordingly. The PMMA plate used for the experiment has an eigenfrequency of 30 KHz. Based on Nyquist’s Theorem, the sampling frequency should be $f_{s}\geq 2\cdot Eigenfrequency$ to capture the wave which is propagated without losing important data. The amount of data, which is acquired, is 5000 per piezo element, thus having 20,000 values per impact to describe the phenomenon.

For the data collection task, a couple of low-cost microcontrollers were tested to evaluate if they are sufficient for the prerequisites of the experiment. The MCUs that have been tested are ESP32 (Espressif Systems, China), Nucleo F303RE (STMicroelectronics, Switzerland) and Arduino Nano 33 BLE (Arduino LLC, Italy). The ESP32 microcontroller was immediately rejected because the max sampling frequency it could achieve was around 20 KHz. This frequency is three times lower than the sampling frequency needed. Despite this fact, the ESP32 in general could provide an elegant solution, considering its dual-core nature. The dual-core architecture could be utilized to separate the model inference and the sensing into independent components, avoiding possible bottlenecks and delays. The STM32 Nucleo F303RE is more than capable to achieve the sampling frequency needed for the data collection. On the other hand, the memory capacity and the core’s speed were not sufficient. The Arduino Nano 33 BLE is the best candidate in comparison with the other options (specs defined in Table 2). First and foremost, it has great compatibility with the TinyML frameworks, which will be discussed later. In addition, it has a great memory capacity to store the amount of data that will be collected and a good enough core speed to create a real-time solution. The core of the MCU is NRF52840 designed by Nordic Semiconductor and the SAADC (successive approximation ADC) can reach up to 200 KHz sampling rate. However, the development process was not problem-free, which was highly expected due to the nature of these low-cost products. These types of MCUs are not designed for specific and demanding experiments.

## 4. Data Collection and Processing

### 4.1. Resource-Constrained Sensing Solution

Every aspect of the sampling procedure is implemented on the hardware level for efficiency. The data is collected using the microcontroller, which sends over the UART bus the sensed values from the PZTs to store them permanently in an offline workstation (e.g., the laptop). The sensors are connected to the analog inputs of the device and the sampling frequency of the analog-to-digital converter (ADC) is set at 100 KHz, to ensure that the impact phenomenon is captured in as much detail as possible. Additionally, the signal that is captured is a shock wave that stimulates the eigenfrequencies of the structure for a small period of time, thus the correct way to treat the captured signal for a specific experiment is to consider it as one instance of 20 k samples (4 sensors × 5 k samples).

The Arduino can achieve up to 200 KHz; however, when sampling a predefined number of samples with that frequency, important information at the end of the impact is not captured. For the signal recording, an auto-trigger mechanism is developed on the device to initiate the acquisition procedure. The primary purpose of the trigger mechanism is to reduce the power consumption and perform only meaningful model inferences on the device (i.e., when an actual impact occurs). This has been achieved by reducing the memory transactions and the processor’s operations during the impact anticipation stage, by buffering 400 samples (100 per sensor). Initially, the MCU samples 400 values, and during the impact anticipation phase 400 samples are continuously acquired. However, only the two most recent batches of 400 samples are stored every time and the only action the processor performs is to check if the relative change in two successive buffered readings (in the last values of the batch) is greater than a threshold set to 10%. Then, if this requirement is met, the impact is detected by preserving the latest batch of 400 samples and sampling the additional 19,600 values.

The collection of the proposed dataset contains a number of challenges, which we outline below, along with their mitigation measures.

- Data quality. This is addressed by executing multiple repetitions of the same unique experiments and defining the optimal impact samples using the Sobol algorithm.
- Reliable impact stimulation methodology. This is addressed by our steel ball drop setup in Figure 4 to ensure consistent labels and reproducible experiments.
- PZT sensor stability. This is addressed by manual testing of each PZT sensor’s responses before including them in the final experiments.
- High-frequency data sampling with automated acquisition and processing of samples. Below, we elaborate on how we addressed the last challenge.

The whole process of the sensors reading is implemented on the hardware level, exploiting the Arduino’s successive approximation ADC (SAADC), and, thus, the processor is not responsible for enabling the analog channels and storing the values in the memory repeatedly. Although SAADC’s sampling rate is over the mark of 15 KHz and operates with multi-channel mode, it is important to note that the Arduino introduces memory writing abnormalities, due to synchronization problems updating SAADC’s storing buffer index and size. Hence, the main solution to overcome this problem is to transfer the data to a second data structure as soon as reading of all the available channels is finished and then restart the SAADC to reinitialize the buffer.

**Figure 7:**
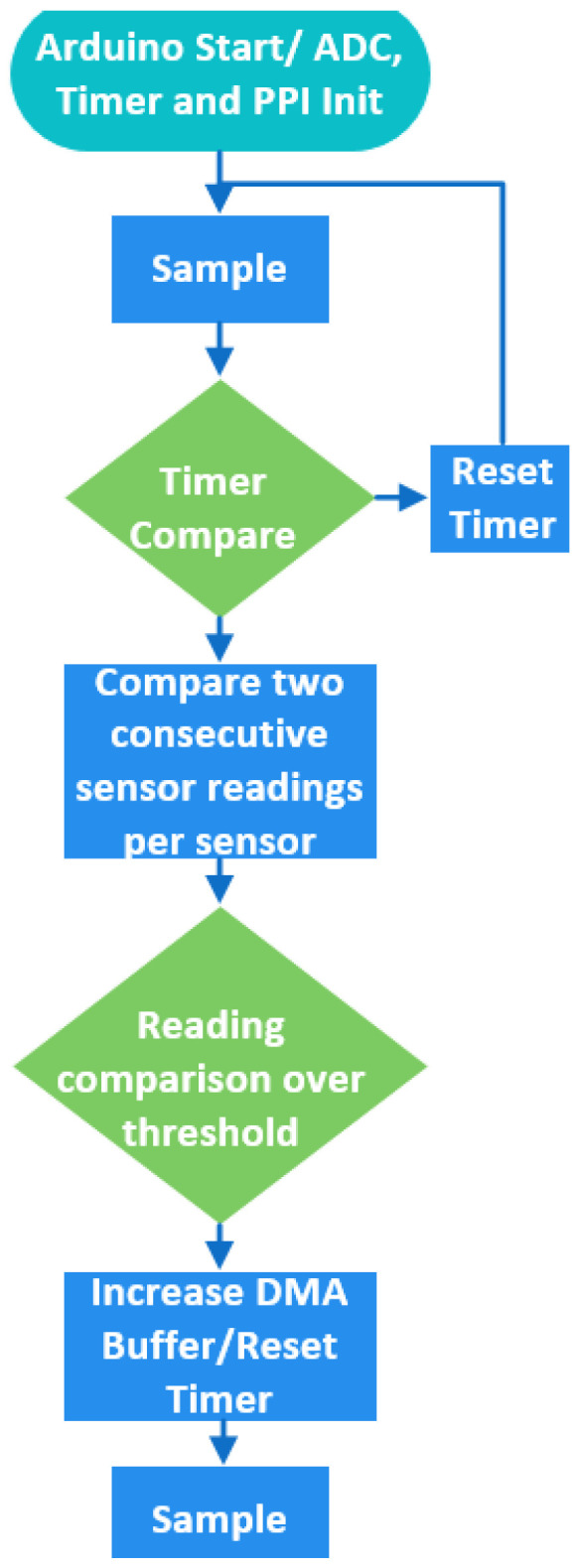
Sampling procedure.

However, we exploit the programmable peripheral interface (PPI), interconnecting the SAADC and a timer to synchronize the needed operations to update the size of the SAADC’s buffer using timer triggers. By using the PPI, the SAADC’s restarting is avoided. By using the PPI, the SAADC’s restarting is avoided. The SAADC’s internal resistance is disabled and the gain is set to $\frac{1}{6}$. The reference voltage is 0.6 Volt. This trigger aspect enables the inferences to run intermittently due to energy limitations, while monitoring the structure. Overall, the sampling latency (setting and initializing the ADC plus storing the data) is 8.5 μs. The sampling procedure is outlined in Figure 7.

### 4.2. Dataset Outline

The data used in this paper is published as the Impact Events dataset [12] (available online: https://zenodo.org/record/7199346 (accessed on 30 December 2022)). Each experiment is denoted by its (x,y,h) coordinates, position (class/tile in the grid) and is repeated at least three times in order to enhance the dataset, but also ensure the robustness of the experiment. The number of acquired samples for each experiment is 5000 samples for each sensor. The final dataset includes 771 instances (multiple repetitions for 159 distinct impacts) and the labels are the corresponding squares $q_{x,y}$ based on the experiment’s (x,y) coordinates. An overview of the dataset’s format is depicted at Table 3.

**Figure 8:**
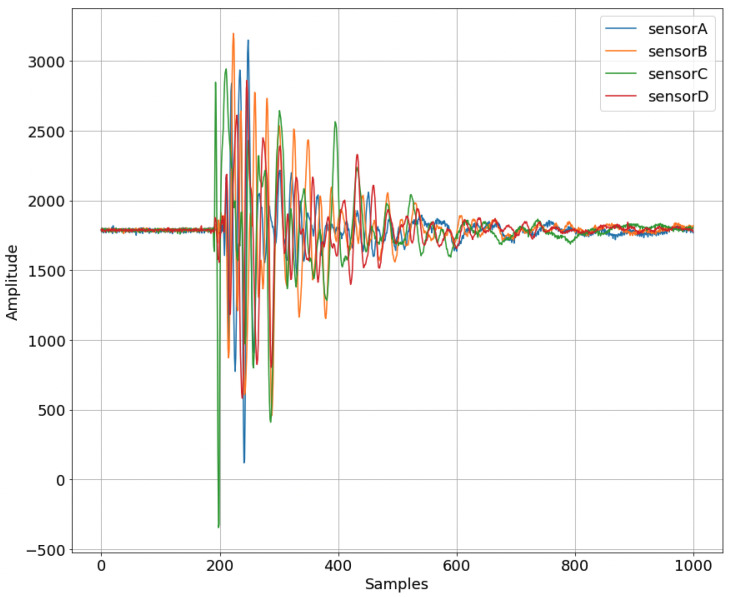
Raw sensor values over the first 1 k samples.

Furthermore, in Table 4 we provide common statistical measures to describe the PZT sensor measurements, while in Figure 8 we present an example of sensors’ response to an impact.

### 4.3. Feature Extraction and Selection

In this work, we experiment with machine learning models that take as input tabular data, and, thus, we need to transform the raw signal data from the four PZT sensors into the corresponding tabular representation. We extract numerous statistical measures, given that they are computationally suitable for the MCU, from the Impact Events dataset. In Table 5, we outline the 32 extracted feature categories that result to a total of 128 features (32 features × 4 sensors) per experiment and our aim is to select an optimal number of features from this set, to speed up the on-device data processing pipeline as well as the ML model size and inference.

Usually, only a subset of features is important and representative of the dataset to result in high accuracy for machine learning models. Therefore, after extracting several statistical features, we select the features, by performing the following steps. First, we calculate the Pearson correlation amongst all the features and eliminate those whose correlation exceeds the threshold of 0.9, in order to keep only uncorrelated features in the dataset. In addition, we remove all the zero-variance, i.e., features that have the same value in all samples.

Afterwards, we calculate the ANOVA (ANOVA is a well-performed and accepted method for feature importance ranking) F-values for the remaining features. In particular, the correlations between the extracted signal features and target columns are calculated by the ANOVA F-test. The results of this test are used for feature selection, where the first five features that are strongly correlated to each of the target variables (*x*-coordinate, *y*-coordinate and fall height) are found. Each F-test evaluates the hypothesis that the response values grouped by predictor variable values are drawn from populations with the same mean against the alternative hypothesis that the population means are not all the same. A small *p*-value of the test statistic indicates that the corresponding predictor is important. The output scores is –log(*p*). Therefore, a large score value indicates that the corresponding predictor is important.

Eventually, we select, examining also their computational complexity, the following feature groups (statistical measures), for each sensor: absolute maximum value, minimum, standard deviation, kurtosis and skewness. Our processed dataset consists of the target feature describing the impact area and thus contains 21 classes/areas, as well as 20 input features in total (5 statistical measures × 4 sensors). For model training, the features are calculated in the offline workstation (e.g., laptop) using the tsfresh library [36] and for on-device model inference, they are implemented in C code in the MCU.

## 5. Machine Learning Models

The proposed solution runs on the extreme edge and in particular on an IoT device, providing real-time information for impact events on the structure, by specifying the area of interest they occurred. Thus, the selected model’s size and inference latency should be as efficient as possible, in order to achieve real-time monitoring, and accurate predictions on the output. The models that have been tested, considering the inference time and memory footprint, are a random forest classifier (RF), XGBoost, support vector machines (SVM) and a shallow neural network. We note that our modeling approach is structure-agnostic, meaning that the impact localization problem is solved in a purely data-driven fashion and independently of the material and geometry of the structure, as the models are trained with the sensor data and do not leverage any physics-based attributes. To train and evaluate the models, the data is split into training and test sets with a 70% and 30%, respectively.

### 5.1. Random Forest

Random forest (RF) is an ensemble of two or more decision trees, called estimators, which focuses on eliminating the disadvantages a decision tree introduces [37]. Such disadvantages are over-fitting and low predictive accuracy. Each tree, which constructs the RF, is obtained by randomly selecting a subset from the dataset and creates the decision tree based on it. Samples of the dataset can be included in more than one subset. Constructing the decision trees with that procedure ensures that the RF consists of uncorrelated trees, reducing the risk of over-fitting, and invariability to outliers and noise. This method is known as bootstrapping. The output that the RF produces is deducted by bootstrap aggregation (bagging). For a classification task, bagging is, essentially, the majority voting of each particular estimator.

The general structure of the RF model is defined by three main hyperparameters, depth of each tree, numbers of estimators and number of sampled data. In general RF’s complexity scales at a large rate when the estimators and depth of each tree increases. However, reducing the initial features by feature engineering, translates to a huge reduction to the needed estimators and depth. Moreover, one major advantage of random forest over neural networks is the explainability aspect, which provides crucial information about the factors that determine each prediction. This is especially important for critical domains such as SHM applications that determine the health status and longevity of the structures.

After experimenting with the hyperparameters of the RF model, the combination that yields an accuracy over 95% comes from the following setting: (i) estimators: 30, (ii) depth: 8, and (iii) sampled Data: 280.

### 5.2. Shallow Neural Network

Overall, the model architecture should have an optimal balance concerning computational complexity and memory occupation, while keeping an acceptable accuracy ratio. In general, there are two main approaches to achieve this [38]. The first is to create a small and sufficient architecture and gradually augment it until the accuracy criteria are met, while the second approach follows the opposite route, i.e., to create a complex and highly accurate DNN or CNN and then use TinyML techniques to optimize the model so it fits on the microcontroller alongside the rest of the operations. As a first step, in the current study we follow the first approach, and implement a shallow neural network (SNN).

Shallow NNs are NNs that consist of one or two hidden layers, although combining them with the selected features can provide a trained model with a low number of parameters and high accuracy. Since, the inputs are the extracted features, which means their scales are different, standardization needs to be applied on the data, otherwise the features are not comparable to one another. If standardization is not applied there are chances of higher weightage to features with higher magnitude. Given that the number of hidden layers is decreased, it ensures that inference time and memory footprint are not prohibitive.

**Figure 9:**
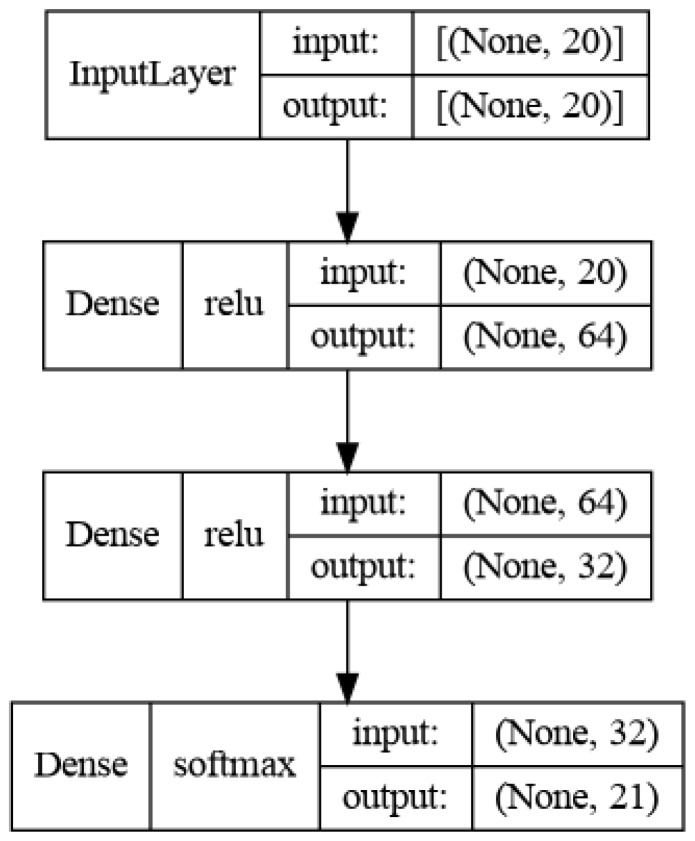
Shallow neural net architecture.

The best architecture that provided the highest accuracy is depicted in Figure 9. The final model consists of 4117 parameters with float 32 precision. The one and only optimization method we applied is quantization with float 16 (the model is denoted later on as Q-SNN), in order to convert the model to an MCU compatible and exploitable form. Quantization is the procedure to reduce the numerical precision, when storing tensors or executing operations. This yields compact models and lower inference time, whereas the model’s accuracy reduction is insignificant and this is due to the low number of parameters at the initial architecture. In addition, one of the main techniques for model reduction is pruning, as it removes weights from neurons, and this results in less parameters and model size. However, the application of pruning methods is avoided in our case as we notice in our experiments a rapid drop in the model’s accuracy.

### 5.3. Gradient Boosted Trees and Support Vector Machines

The gradient boosted trees model is an ensemble of decision trees, like random forest. The individual trees are created in a sequential manner and the idea behind these trees is to combine individual trees with high loss (weak learners), in order to create a decision tree that minimizes the loss function (strong learner). The goal of each tree is to minimize the error of the previous tree. At each iteration of the algorithm, a decision tree is appended, until there are no further improvements. Gradient Boosted Trees (GBTs) is one of the most capable machine learning algorithms; however, it is prone to over-fitting, and the memory footprint and the inference time is high, due to the size of the ensemble and the hyperparameters that need to be stored. In this paper, we experiment with XGBoost, 1.7.0 which is one of the fastest implementations of GBTs. It does this by tackling one of the major inefficiencies of gradient-boosted trees: considering the potential loss for all possible splits to create a new branch (especially in the case where there are thousands of features, and therefore thousands of possible splits). XGBoost tackles this inefficiency by looking at the distribution of features across all data points in a leaf and using this information to reduce the search space of possible feature splits.

Support vector machines (SVM) is a statistical learning model that aims to provide the best margins between the discrete classes, in order to classify the data, whereas when used for regression it provides the best margins that encapsulate the data. When used for high dimensional data, SVMs map the data to hyperplanes to provide the hyperplane with the largest separation of the data points. To achieve the data transformation, SVM uses kernel functions. SVM is very sensitive to noise and is difficult to interpret the final model. A first assumption is that SVM and XGBoost models will not be optimal solutions in our context due to their aforementioned disadvantages. However, we opt to benchmark them as they are one of the most commonly used and effective algorithms for tabular data [39] and thus they provide a comparison basis for our case.

### 5.4. On-Device Model Deployment

To realize the on-device ML model operation, we first train the models in our personal workstation and then deploy them in the Arduino to perform the inference. Concerning the actual model conversion in an appropriate format for the MCU, there is not any single library that can be used for both the traditional ML algorithms and the neural networks.

In specifics, to convert the former models (RF, XGBoost, SVM), we use the micromlgen library [40]. This library converts the first two models into C++ header file using multiple *IF-ELSE* statements to represent the decision graph and for the SVM conversion it also produces a C++ header file that includes the needed kernel operations. Concerning the SNN, the TensorFlow Lite (TFLite) library [41] is utilized, which introduces quantization, pruning and several other optimizations methods. TFLite converts the initial model to an optimized FlatBuffer format, and the optimizations occur on the converted model. After the model is in TFLite form, a C++ header file is generated with the hex representation of the model by using xxd command on Unix. This header file is later manipulated by the TensorFlow Lite Micro C++ API on the microcontroller to invoke the inference.

Finally, to calculate the footprint metrics, the model size for RF, XGBoost, SVM and SNN corresponds to the size of the joblib file, since no optimizations occur or size reduction, and .tflite file, respectively. In addition, for the inference (prediction) time of each model we average the inference timings over the test set instances.

## 6. Evaluation and Discussion

To evaluate the models, we focus on quality metrics to assess the models’ accuracy and footprint metrics to assess the on-device model performance. For the footprint metrics we benchmark the models deployed in the Arduino based on their model size, the model inference time for one single instance (impact location prediction), the inference time plus the time needed to perform the feature extraction (FE), the RAM occupation during inference and feature extraction, the Flash memory occupation for storing the model and the associated C code and, lastly, the energy consumption during these operations. Concerning the quality evaluation metrics, we use classification metrics such as accuracy, Matthews correlation coefficient (MCC), F1-Score, precision and recall, which are defined as follows:

(2) $$MCC=\frac{TP*TN-FP*FN}{\sqrt{\left(TP+FP)(TP+FN)(TN+FP)(TN+FN\right)}}$$

(3) $$Accuracy=\frac{TP+TN}{TP+TN+FP+FN}$$

(4) $$F1\;score=2*\frac{Precision*Recall}{Precision+Recall}$$

(5) $$Precision=\frac{TP}{TP+FP}$$

(6) $$Recall=\frac{TP}{TP+FN}$$

where TP, TN are the true positives and negatives and FP, FN the false positives and negatives predicted outcomes of the model.

- *MCC* is a perfectly symmetric metric that represents the correlation between true values and predicted ones. Similar to Pearson’s correlation coefficient, it ranges from −1 to 1. A score of 1.0 means a perfect classifier, while a value close to 0 means the classifier is no better than random chance.
- *Accuracy* is intuitively the overall fraction of predictions the model got right. However, it is not enough to evaluate the models using only this metric, especially for class-imbalanced datasets.
- *F1-Score* is a measure of a test’s accuracy. It provides a way to combine both precision and recall into a single measure that captures both properties.
- *Precision* represents the proportion of positive identifications that were actually correct.
- *Recall* represents the proportion of the actual positives that were identified correctly.

**Figure 10:**
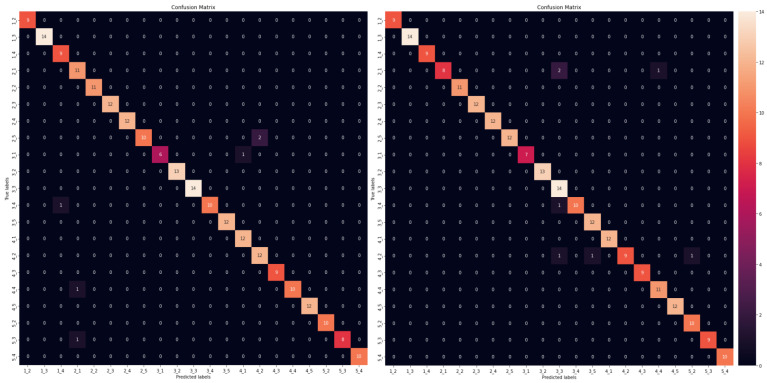
Confusion matrices for random forest (**left**) and shallow neural network (**right**).

The results are summarized in Table 6 for quality metrics and Table 7 for footprint metrics, while Figure 10 depicts the performance of the models for the localization problem in the 5 × 5 grid case for both RF and Q-SNN models. The tables are grouped by the grid cases, i.e., the number of classes in the multi-class classification problem, without considering the four corner classes which include each sensor, respectively, due to noisy signals. Thus, based on the 5 × 5 grid description in Section 3.1, the 5 × 5 grid case results in 21 classes (square tiles in the grid), while the 4 × 4 and 6 × 6 grids result in 12 and 32 classes, respectively. In our initial work [42], we studied the multiclass classification problem only with the 21 classes (5 × 5 plate grid); however, in this paper we also increased and decreased the discretization of the plate and further studied the 4 × 4 and 6 × 6 grid cases. The rationale behind this analysis was to examine the impact on the models’ performance and thus prove their robustness for the given problem, by solving different types of classification–impact localization. In addition, we provide the arithmetic mean and standard deviation results for all the metrics, to prove the statistical smoothness of our approach. These have been calculated for all the models across all the grid cases, meaning that we have trained and evaluated the machine learning models for n=10 times and evaluated the classification (quality) and performance (footprint) metrics. To calculate the latter, all the models were re-deployed on-device in the Arduino for each case.

In Table 6 and Table 7, we have highlighted in bold the best model per grid case and we notice that, in general, all the models have similar and satisfactory performance across the metrics. Examining the average and standard deviation of the results, we can tell that the models are robust and stable in their predictions since they have very small standard deviations (across their multiple training cycles). We conclude that the best model to be selected is the RF, as it has the best overall performance in the quality and footprint metrics. The Q-SNN comes second, with very close results in all the metrics except the Flash memory, which is on average three times larger than the RF. In addition, another advantageous aspect of RF is the explainable insights by design (feature importance) which is an important trustworthiness criterion for real-time industrial applications supported by embedded intelligence, as the one we study in this paper. In addition, we observe that in this problem the nature of the data results in higher classification accuracy in the 6 × 6 grid case which has the most classes (32). This is due to the fact that by increasing the cell area (and decreasing this way the number of classes) more, less correlated, impacts are included since they have greater distance between them and, thus, the models’ capability to distinguish between the classes is less effective.

Despite the timing differences between the models, the inference time of all models is still acceptable for the requirements of the studied task (which is application-dependable). Additionally, the memory demand of RF and Q-SNN models is very low and insignificant considering the specifications of our MCU. In fact, the present models can be also fitted in even more resource constrained MCUs. However, the XGBoost model has a huge memory footprint, that makes it unfeasible to deploy on the MCU and benchmark the inference timings as well as the RAM and Flash memory occupation. Similarly, the SVM model is very large in this context but it can be deployed on the MCU, although it is not optimal with respect to MCU’s available memory for other data storing operations. Concerning the optimization of the shallow NN with the quantization method, we obtain remarkable results, mainly in the reduction of the inference time but with a very small drop in the accuracy. Regarding energy consumption, when the Arduino is idle it consumes between 18 and 19 mAh, due to the data acquisition and sampling process that buffers temporary values until the trigger is enabled (see Section 4.1). However, when the Arduino performs the models’ inference, the consumption ranges between 22.4 and 23.4 mAh.

We also wish to note that the high accuracy scores are achieved by using only 540 instances (experimental impacts) for training the models (the rest 231 instances belong to the test set). This proves that the proposed design of experiment, which defines the impact experiments to be executed using the Sobol algorithm, results in a representative data space for the given problem and, thus, contributes to the high ML model accuracy. According to the results and our experience, using feature engineering and traditional ML practices is a useful strategy towards real-time TinyML-based IoT systems, even though it is (justifiably, for the known reasons) not the most popular approach in the literature, especially for TinyML research. Our rationale, is to examine all possible solutions and keep the simplest and most effective ones. We suggest following the aforementioned approach depending on the application and data requirements, complimentary with ANN modeling and exploration. Indeed, in the future we intend to experiment more with ANNs (using raw signal data without features as well), given their inherent potential to reach optimal architectures with the highest accuracy and smallest footprint, by leveraging optimizations methods such as pruning, quantization, knowledge distillation, etc., whereas conventional ML models have low potential to be optimized when they are already large in size.

Lastly, concerning the comparison with the state-of-the-art, there are limited works with polymer composites as they represent specific challenges due to their susceptibility to impact damage. In addition, it is not quite feasible to directly compare our methodology to others as they deal with different structures and materials or even sensors. In addition, there are no public datasets except ours so that researchers can directly benchmark their models. However, our models’ results are comparable to [18], which studies impact localization problems with classification and reports 94.3% average prediction accuracy. Similarly, ref. [17] reached accuracy values between 94.3% and 100% when predicting distinct impacts on similar locations that their models had been trained with. We, therefore, state that our models achieve comparable accuracy and they are probably more resource-efficient and we demonstrate this through a material agnostic methodology.

## 7. Conclusions

In this paper, we aim to integrate effectively the characteristic resource-constrained nature of sensors into an industrial application characterized by strict real-time specifications. In particular, we present a low-cost, resource-frugal IoT solution powered by TinyML technology to solve the impact localization problem of a thin PMMA plate. We developed and configured the IoT hardware, we conducted feature engineering and data processing, and finally delivered two models that enable on-device impact localization services. We also share our dataset to facilitate researchers in studying impact detection and localization in SHM applications (applying both supervised and unsupervised ML techniques), as our search for available experimental data did not lead us to any result that could serve our objectives. The best results come from the random forest model, which achieves a classification accuracy that ranges from 93 to 98% across the grid cases and on-device footprint as low as 333 ms for executing the inference (including the data processing and feature extraction process), 78–88 KB and 162 KB for Flash and RAM memory consumption respectively. These results motivate the concept of our implementation for real-time, embedded intelligence in SHM applications and indicate promising potential for further exploration in SHM-related problems and associated TinyML research.

Concerning the limitations of this study, we acknowledge the potential limits of Arduino’s maximum sampling frequency capability (up to 200 KHZ) that may pose restrictions in cases where a higher sampling rate might be required. Moreover, other factors such as (a) the data processing and ML modeling parts have been validated only in one PMMA plate which has specific geometry and properties, (b) the impact events come from a single source (steel ball) that produces specific excitation due to its mass and geometry and (c) the quality of PZT sensors, might potentially affect the generalizability of the proposed methodology in different scenarios.

In the future, we plan to extend our dataset by conducting experiments with more types of impact sources and more setups of the same plate and sensors (as they are not identical and discrepancies may exist). In addition, we intend to experiment with additional modeling techniques such as convolutional neural networks with the raw PZT data as well as generate numerical simulation data and explore the benefits of utilizing hybrid data for the given problem. Lastly, we consider exploring the application of signal processing techniques for data cleaning and preprocessing as well as extracting additional features such as the time of arrival (ToA), i.e., the time that it takes for the in-plane strain waves, generated by the impact, to arrive at the sensors.

## Figures and Tables

**Table 1:** Specifications of Ceramic piezoelectric transducer CEB-35D26.

Parameter	Units
operating voltage	max. 30 V
resonant frequency	typ. 2.6 Hz
weight	max. 2.0 g
dimensions	Ø35 × 0.53 mm

**Table 2:** MCU specifications.

Arduino NANO 33 BLE Specifications	
Microcontroller	nRF52840
Clock Speed	64 MHz
Flash Memory	1 MB
SRAM	256 KB
Analog Input Pins	8
ADC Max Sampling Rate	200 ksps
ADC Resolution	12 bit

**Table 3:** Dataset format.

SensorA	SensorB	SensorC	SensorD	sampleNo	typeofimpact	*x*	*y*	height	position	ID

**Table 4:** Descriptive statistics for the four PZT sensor measurements.

Statistic	SensorA	SensorB	SensorC	SensorD
count	3,855,000	3,855,000	3,855,000	3,855,000
mean	1790.466	1794.333	1793.815	1792.419
std	86.822	101.574	83.672	100.586
min	−357	−385	−352	−371
25%	1775	1781	1780	1782
50%	1796	1799	1799	1797
75%	1810	1812	1812	1809
max	4048	4057	4040	4055

**Table 5:** List of all the extracted features.

Feature	Description
Energy	Energy of the time series
Absolute maximum	Highest absolute value
Absolute sum of changes	Sum of absolute values of consecutive changes
C3	Measurement of nonlinearity
Count above mean	Number of values greater than the mean
Count below mean	Number of values lesser than the mean
Energy ratio by chunks	Ratio of the *i*-th chunk’s energy over the energy of N chunks
First location of maximum	Index of the maximum value’s first appearance relative to the length of the time series
First location of maximum	Index of the minimum value’s first appearance relative to the length of the time series
Kurtosis	Signal’s tails measurement relative to normal distribution
Large standard deviation	Check if the standard deviation is *r* times greater than max−min
Longest strike above mean	Number of consecutive values above mean
Longest strike below mean	Number of consecutive values below mean
Maximum	Highest value
Mean	Mean of the time series
Mean of absolute change	Mean value of absolute change
Mean change	Average of differences of subsequent values
Median	Median of the time series
Minimum	Lowest value
Skewness	Calculates the lack of symmetry
Standard Deviation	Measurement of time series’s dispersion relative to the mean
Symmetry looking	Checks the symmetry of the time series
Variance	Variability of the time series
Variance larger than standard deviation	Checks if the variance is greater than standard deviation

**Table 6:** ML models’ quality evaluation metrics. The results from the best model per grid case are highlighted in bold.

Grid	Model	Accuracy	F1 Score	MCC	Precision	Recall
4 × 4	RF	**0.93 ± 0.05**	**0.925 ± 0.03**	**0.932 ± 0.04**	**0.945 ± 0.02**	**0.925 ± 0.01**
	SVM	0.905 ± 0.11	0.88 ± 0.10	0.903 ± 0.09	0.91 ± 0.10	0.888 ± 0.10
	XGBoost	0.926 ± 0.14	0.925 ± 0.10	0.923 ± 0.11	0.934 ± 0.15	0.921 ± 0.13
	SNN	0.926 ± 0.10	0.923 ± 0.10	0.925 ± 0.11	0.93 ± 0.09	0.938 ± 0.09
	Q-SNN	0.922 ± 0.13	0.894 ± 0.13	0.921 ± 0.11	0.903 ± 0.11	0.907 ± 0.12
5 × 5	RF	0.922 ± 0.09	0.91 ± 0.09	0.92 ± ± 0.08	0.9 ± 0.08	0.916 ± 0.07
	SVM	0.909 ± 0.18	0.89 ± 0.20	0.90 ± 0.19	0.93 ± 0.17	0.898 ± 0.17
	XGBoost	0.9 ± 0.11	0.87 ± 0.11	0.89 ± 0.10	0.877 ± 0.11	0.875 ± 0.10
	SNN	0.932 ± 0.08	0.934 ± 0.08	0.930 ± 0.07	0.947 ± 0.06	0.93 ± 0.06
	Q-SNN	**0.949 ± 0.14**	**0.942 ± 0.13**	**0.947 ± 0.14**	**0.948 ± 0.10**	**0.95 ± 0.10**
6 × 6	RF	**0.981 ± 0.10**	**0.981 ± 0.10**	**0.98 ± 0.11**	**0.983 ± 0.09**	**0.98 ± 0.08**
	SVM	0.90 ± 0.11	0.901 ± 0.11	0.90 ± 0.12	0.91 ± 0.12	0.907 ± 0.12
	XGBoost	0.926 ± 0.16	0.925 ± 0.15	0.923 ± 0.16	0.93 ± 0.13	0.921 ± 0.12
	SNN	0.979 ± 0.14	0.977 ± 0.16	0.978 ± 0.16	0.984 ± 0.12	0.974 ± 0.13
	Q-SNN	0.968 ± 0.06	0.968 ± 0.006	0.967 ± 0.008	0.976 ± 0.004	0.965 ± 0.007

**Table 7:** On-device footprint evaluation metrics. The results from the best model per grid case are highlighted in bold.

Grid	Model	Inference (ms)	Inf. + FE (ms)	Model Size (KB)	RAM (KB)	Flash (KB)
4 × 4	RF	**0.086 ± 0.0067**	**333.85 ± 0.96**	**15**	**162**	**88**
	SVM	73.614 ± 0.15	394.552 ± 0.46	360	162	836
	XGBoost	-	-	4004	-	-
	SNN	0.894 ± 0.02	374.36 ± 0.2	24	202	257
	Q-SNN	0.254 ± 0.03	318.66 ± 0.03	16	192	247
5 × 5	RF	**0.084 ± 0.0049**	**333.87 ± 0.98**	**12**	**162**	**82**
	SVM	65.827 ± 0.15	374.662 ± 0.5	227	162	541
	XGBoost	-	-	2457	-	-
	SNN	0.898 ± 0.01	377.27 ± 0.1	20	199	254
	Q-SNN	0.259 ± 0.03	320.67 ± 0.032	16	192	254
6 × 6	RF	**0.084 ± 0.0049**	**333.87 ± 0.98**	**8**	**162**	**78**
	SVM	54.267 ± 0.10	376.892 ± 0.47	168	162	420
	XGBoost	-	-	835	-	-
	SNN	0.911 ± 0.01	379.27 ± 0.05	20	199	254
	Q-SNN	0.284 ± 0.08	318.091 ± 0.084	12	190	245

## Data Availability

Ioannis Katsidimas, Thanasis Kotzakolios, Sotiris Nikoletseas, Stefanos H. Panagiotou, Konstantinos Timpilis, and Constantinos Tsakonas (2022). Dataset: Impact Events for Structural Health Monitoring of a Plastic Thin Plate [https://zenodo.org/record/7199346 (accessed on 30 December 2022)].

## References

[1] Dutta D.L., Bharali S. (2021). TinyML Meets IoT: A Comprehensive Survey. Internet Things.

[2] Cawley P. (2018). Structural health monitoring: Closing the gap between research and industrial deployment. Struct. Health Monit..

[3] Goyal D., Pabla B. (2016). The Vibration Monitoring Methods and Signal Processing Techniques for Structural Health Monitoring: A Review. Arch. Comput. Methods Eng..

[4] Malekloo A., Ozer E., AlHamaydeh M., Girolami M. (2022). Machine learning and structural health monitoring overview with emerging technology and high-dimensional data source highlights. Struct. Health Monit..

[5] Shen J., Li Z., Cheng R., Luo Q., Luo Y., Chen Y., Chen X., Sun Z., Huang S. (2014). Eu^3+^-Doped NaGdF4 Nanocrystal Down-Converting Layer for Efficient Dye-Sensitized Solar Cells. ACS Appl. Mater. Interfaces.

[6] Hammam M., El-Mansy M., El-Bashir S., El-Shaarawy M. (2007). Performance evaluation of thin-film solar concentrators for greenhouse applications. Desalination.

[7] Schissel P., Jorgensen G., Kennedy C., Goggin R. (1994). Silvered-PMMA reflectors. Sol. Energy Mater. Sol. Cells.

[8] Blanco N., Gamstedt E., Asp L., Costa J. (2004). Mixed-mode delamination growth in carbon–fibre composite laminates under cyclic loading. Int. J. Solids Struct..

[9] Choi K., Chang F.K. (1994). Identification of Foreign Object Impact in Structures Using Distributed Sensors. J. Intell. Mater. Syst. Struct..

[10] Filios G., Katsidimas I., Nikoletseas S., Tsenempis I. (2019). A Smart Energy Harvesting Platform for Wireless Sensor Network Applications. Information.

[11] Sotiriadis G., Kotzakolios T., Kostopoulos V., Gemou M. Digital Twin Assisted and Embedded Strain Gauge Monitoring System. Proceedings of the Transport Research Arena.

[12] Katsidimas I., Kotzakolios T., Nikoletseas S., Panagiotou S.H., Timpilis K., Tsakonas C. Dataset: Impact events for Structural Health Monitoring of a thin plate. Proceedings of the 20th ACM Conference on Embedded Networked Sensor Systems.

[13] Dipietrangelo F., Nicassio F., Scarselli G. (2023). Structural Health Monitoring for impact localisation via machine learning. Mech. Syst. Signal Process..

[14] Balasubramanian P., Kaushik V., Altamimi S.Y., Amabili M., Alteneiji M. (2022). Comparison of neural networks based on accuracy and robustness in identifying impact location for structural health monitoring applications. Struct. Health Monit..

[15] Datta A., Augustin M.J., Gupta N., Viswamurthy S.R., Gaddikeri K.M., Sundaram R. (2019). Impact Localization and Severity Estimation on Composite Structure Using Fiber Bragg Grating Sensors by Least Square Support Vector Regression. IEEE Sens. J..

[16] Hesser D.F., Kocur G.K., Markert B. (2020). Active source localization in wave guides based on machine learning. Ultrasonics.

[17] Tabian I., Fu H., Sharif Khodaei Z. (2019). A Convolutional Neural Network for Impact Detection and Characterization of Complex Composite Structures. Sensors.

[18] Jung K.C., Chang S.H. (2021). Advanced deep learning model-based impact characterization method for composite laminates. Compos. Sci. Technol..

[19] Karmakov S., Aliabadi M.H.F. (2022). Deep Learning Approach to Impact Classification in Sensorized Panels Using Self-Attention. Sensors.

[20] Zonzini F., Romano F., Carbone A., Zauli M., De Marchi L. Enhancing vibration-based structural health monitoring via edge computing: A tiny machine learning perspective. Proceedings of the Quantitative Nondestructive Evaluation. American Society of Mechanical Engineers.

[21] Crocioni G., Pau D., Delorme J.M., Gruosso G. (2020). Li-Ion Batteries Parameter Estimation With Tiny Neural Networks Embedded on Intelligent IoT Microcontrollers. IEEE Access.

[22] Athanasakis G., Filios G., Katsidimas I., Nikoletseas S., Panagiotou S.H. TinyML-based approach for remaining useful life Prediction of Turbofan Engines. Proceedings of the 27th International Conference on Emerging Technologies and Factory Automation (ETFA).

[23] Ren H., Anicic D., Runkler T.A. TinyOL: TinyML with Online-Learning on Microcontrollers. Proceedings of the 2021 International Joint Conference on Neural Networks (IJCNN).

[24] Bratu D.V., Ilinoiu R.Ş.T., Cristea A., Zolya M.A., Moraru S.A., Maglogiannis I., Iliadis L., Macintyre J., Cortez P. (2022). Anomaly Detection Using Edge Computing AI on Low Powered Devices. Artificial Intelligence Applications and Innovations.

[25] Funk F., Bucksch T., Mueller-Gritschneder D., Gama J., Pashami S., Bifet A., Sayed-Mouchawe M., Fröning H., Pernkopf F., Schiele G., Blott M. (2020). ML Training on a Tiny Microcontroller for a Self-adaptive Neural Network-Based DC Motor Speed Controller. Proceedings of the IoT Streams for Data-Driven Predictive Maintenance and IoT, Edge, and Mobile for Embedded Machine Learning.

[26] Azimi M., Eslamlou A.D., Pekcan G. (2020). Data-driven structural health monitoring and damage detection through deep learning: State-of-the-art review. Sensors.

[27] Aabid A., Parveez B., Raheman M.A., Ibrahim Y.E., Anjum A., Hrairi M., Parveen N., Mohammed Zayan J. (2021). A Review of Piezoelectric Material-Based Structural Control and Health Monitoring Techniques for Engineering Structures: Challenges and Opportunities. Actuators.

[28] De Oliveira M.A., Monteiro A.V., Vieira Filho J. (2018). A new structural health monitoring strategy based on PZT sensors and convolutional neural network. Sensors.

[29] Bechhoefer E. (2018). Data set for Wind Turbine High-Speed Bearing Prognosis example in Predictive Maintenance Toolbox. https://github.com/mathworks/WindTurbineHighSpeedBearingPrognosis-Data.

[30] Figueiredo E., Park G., Figueiras J., Farrar C., Worden K. (2009). Structural Health Monitoring Algorithm Comparisons Using Standard Data Sets.

[31] Teloli R.d.O., da Silva S. (2019). A new way for harmonic probing of hysteretic systems through nonlinear smooth operators. Mech. Syst. Signal Process..

[32] Marzani A., Testoni N., De Marchi L., Messina M., Monaco E., Apicella A. (2020). An open database for benchmarking guided waves structural health monitoring algorithms on a composite full-scale outer wing demonstrator. Struct. Health Monit..

[33] (2020). CUI Devices. CEB-35D26 Datasheet. https://gr.mouser.com/datasheet/2/670/ceb_35d26-1776373.pdf.

[34] Bratley P., Fox B.L. (1988). Algorithm 659: Implementing Sobol’s Quasirandom Sequence Generator. ACM Trans. Math. Softw..

[35] Jiriwibhakorn S., Coonick A. Fast critical clearing time estimation of a large power system using neural networks and Sobol sequences. Proceedings of the 2000 Power Engineering Society Summer Meeting (Cat. No.00CH37134).

[36] Christ M., Braun N., Neuffer J., Kempa-Liehr A.W. (2018). Time series feature extraction on basis of scalable hypothesis tests (tsfresh— A python package). Neurocomputing.

[37] Breiman L. (2001). Random forests. Mach. Learn..

[38] Menghani G. (2021). Efficient Deep Learning: A Survey on Making Deep Learning Models Smaller, Faster, and Better. arXiv.

[39] Shwartz-Ziv R., Armon A. (2022). Tabular data: Deep learning is not all you need. Inf. Fusion.

[40] Eloquentarduino (2020). Micromlgen Library Repository. https://github.com/eloquentarduino/micromlgen.

[41] David R., Duke J., Jain A., Janapa Reddi V., Jeffries N., Li J., Kreeger N., Nappier I., Natraj M., Wang T. TensorFlow Lite Micro: Embedded Machine Learning for TinyML Systems. Proceedings of the Machine Learning and Systems.

[42] Katsidimas I., Kotzakolios T., Nikoletseas S., Panagiotou S.H., Tsakonas C. Smart Objects: Impact localization powered by TinyML. Proceedings of the 20th ACM Conference on Embedded Networked Sensor Systems.

